# Supplementary material for: MuSE: accounting for tumor heterogeneity using a sample-specific error model improves sensitivity and specificity in mutation calling from sequencing data
Source: Genome Biol. 2016 Aug 24;17(1):178. doi: 10.1186/s13059-016-1029-6 (PMC4995747; doi:10.1186/s13059-016-1029-6)
Supplement: Additional file 1 — Table/figure. (PDF 2826 kb) [file 13059_2016_1029_MOESM1_ESM.pdf]

Supplementary Tables

Supplementary Table 1: Number of positive spike-in conditions for the virtual-tumor benchmarking approach. Tumor data coverage varies from 10× to 60× and spike-in VAFs are 0.05, 0.1, 0.2 and 0.4.

Spike-in VAF	Tumor Data Coverage					
	10x	20x	30x	40x	50x	60x
0.05	608,275	608,403	608,438	608,469	608,482	608,486
0.1	608,274	608,403	608,431	608,444	608,446	608,400
0.2	608,274	608,374	608,328	608,172	607,497	605,323
0.4	608,251	608,088	605,272	586,219	516,971	379,905

Supplementary Table 2: Allocation of MuSE and Call-A calls for the ACC data. Calls are categorized by whether they are selected for deep sequencing validation and whether they are unique for one caller or shared by two callers. The number of validated calls is shown in parentheses.

	MuSE				Caller-A			
	Inside Validation Design		Outside Validation Design		Inside Validation Design		Outside Validation Design	
	Unique	Shared	Unique	Shared	Unique	Shared	Unique	Shared
PASS	11 (8)	125 (125)	141	8,900				
Tier-1	39 (7)	111 (99)	221	472				
Tier-2	34 (5)	29 (25)	345	109	121 (30)	290 (268)	1,693	9,584
Tier-3	25 (8)	17 (12)	494	52				
Tier-4	30 (7)	8 (7)	1,102	51				
Total	139 (35)	290 (268)	2,303	9,584	121 (30)	290 (268)	1,693	9,584

Supplementary Figures

Venn Diagrams of MuSE and MuTect Calls Using the Virtual-tumor Benchmarking Data

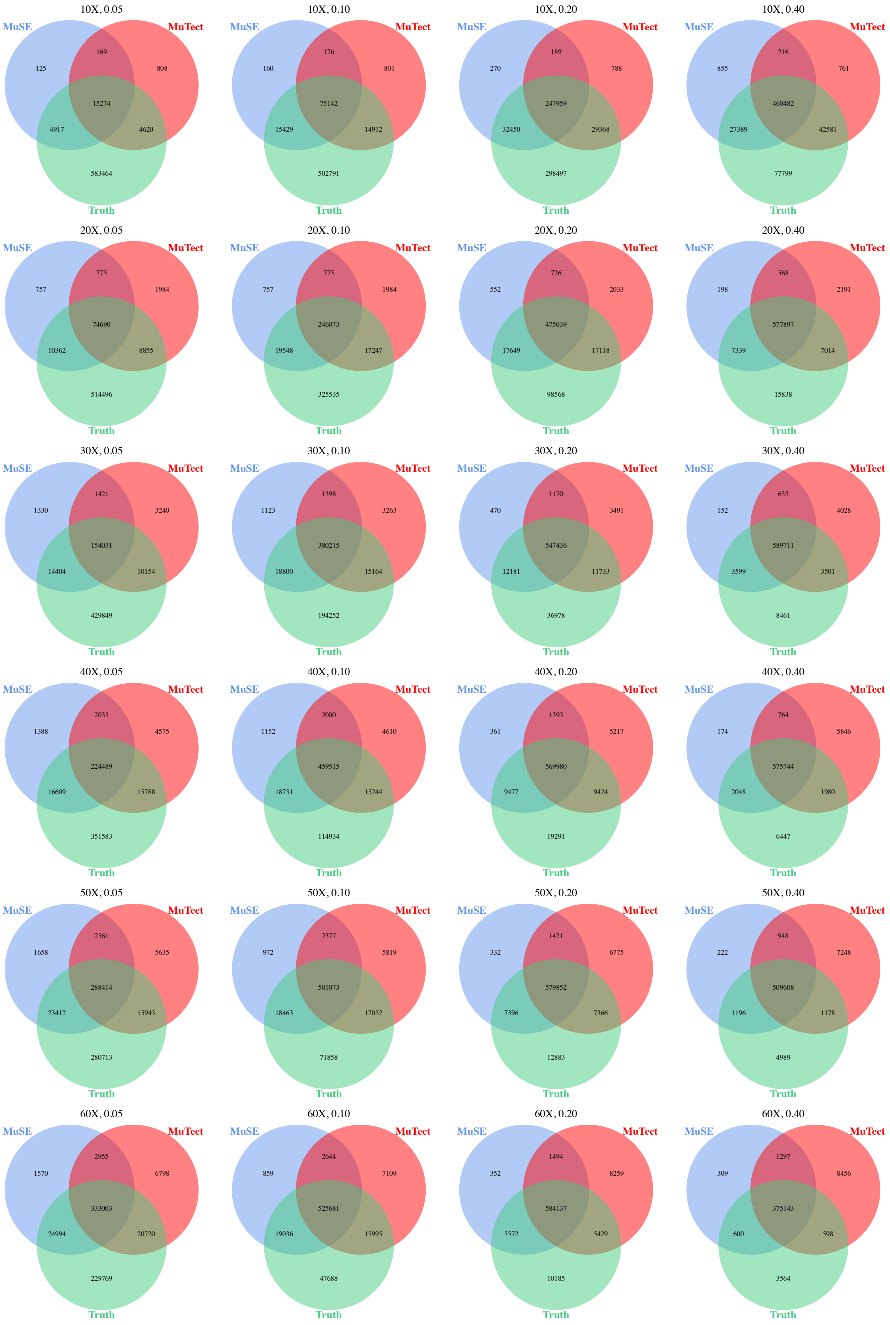

Supplementary Figure 1: Venn diagrams of MuSE and MuTect calls compared with the truth across all combinations of tumor data coverage and VAFs for the virtual-tumor benchmarking data.

Venn Diagrams of Calls from Five Different Callers Using the ACC Data

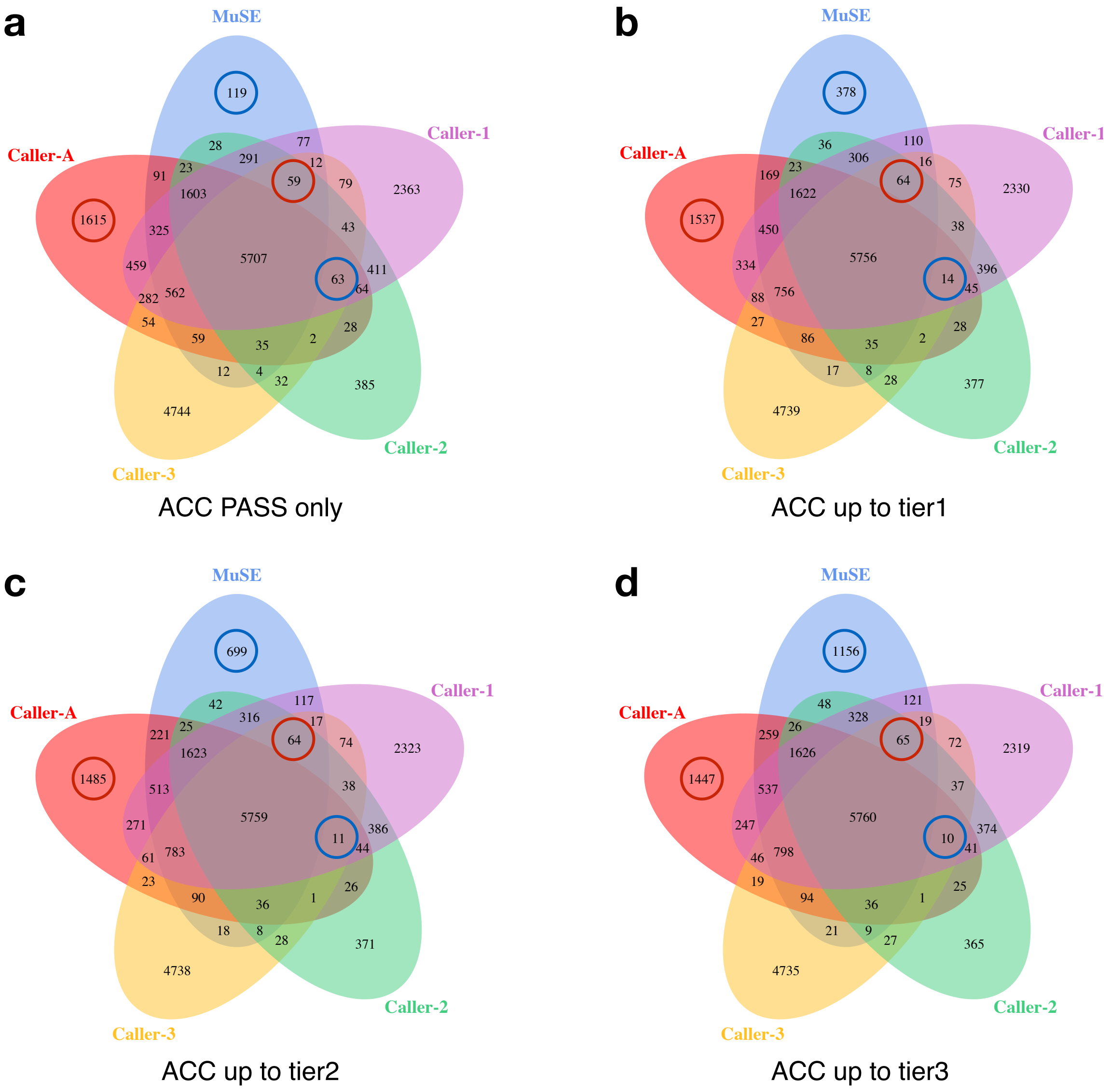

Supplementary Figure 2: Venn diagrams of calls from five different callers using the ACC data. All the calls except those of MuSE are extracted from TCGA mutation annotation format (MAF) file. MuSE calls from different cutoffs (PASS only, up to Tier-1, up to Tier-2 and up to Tier-3) are used. The blue circles label the number of MuSE unique calls and the number of calls missed by MuSE but captured by the other four callers. The red circles have the same meaning but apply to Caller-A.

Density Distributions of $\log\left(2\pi_{\text{somatic,tumor}}\right)$ from MuSE on the Virtual-tumor Benchmarking Data

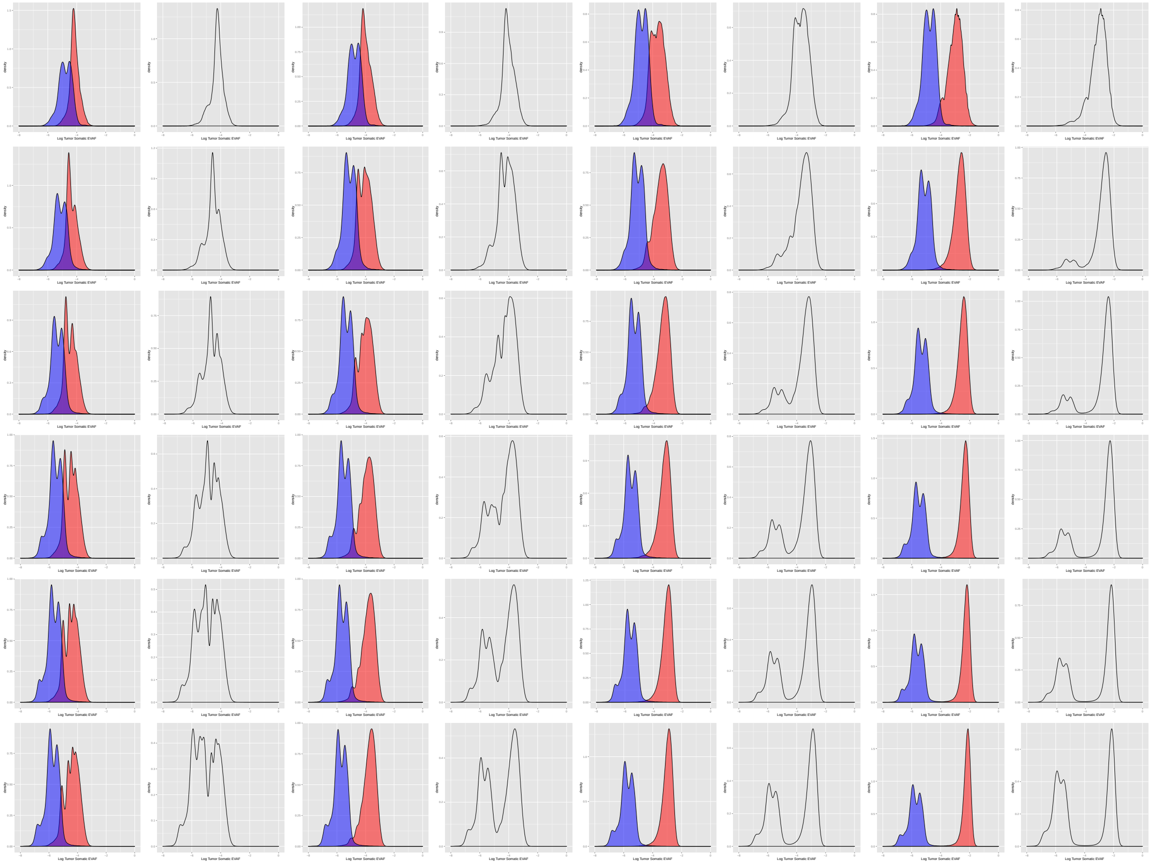

Supplementary Figure 3: Density distributions of $\log\left(2\pi_{\text{somatic,tumor}}\right)$ from MuSE on the virtual-tumor benchmarking data. Rows correspond to different coverage depth, from $10\times$ to $60\times$, at an increment of $10\times$. Every two columns correspond to a different scenario for VAF, at 0.05, 0.1, 0.2 and 0.4, from left to right. In each pair of columns, the panel on the left shows the density of true mutations (in red) and that of the remaining reference positions (in blue), respectively. The panel on the right shows the density of all positions together without the knowledge of mutation status.
